# Erythroid-Specific Expression of β-globin from *Sleeping Beauty-*Transduced Human Hematopoietic Progenitor Cells

**DOI:** 10.1371/journal.pone.0029110

**Published:** 2011-12-28

**Authors:** Lucas M. Sjeklocha, Chang-Won Park, Phillip Y-P Wong, Mark J. Roney, John D. Belcher, Dan S. Kaufman, Gregory M. Vercellotti, Robert P. Hebbel, Clifford J. Steer

**Affiliations:** 1 Division of Gastroenterology, Hepatology and Nutrition, Department of Medicine, University of Minnesota Medical School, Minneapolis, Minnesota, United States of America; 2 Department of Genetics, Cell Biology and Development, University of Minnesota, Minneapolis, Minnesota, United States of America; 3 Vascular Biology Center, University of Minnesota Medical School, Minneapolis, Minnesota, United States of America; 4 Division of Hematology, Oncology and Transplantation, Department of Medicine, University of Minnesota Medical School, Minneapolis, Minnesota, United States of America; 5 Stem Cell Institute, University of Minnesota Medical School, University of Minnesota, Minneapolis, Minnesota, United States of America; University of Sao Paulo – USP, Brazil

## Abstract

Gene therapy for sickle cell disease will require efficient delivery of a tightly regulated and stably expressed gene product to provide an effective therapy. In this study we utilized the non-viral *Sleeping Beauty* (*SB*) transposon system using the *SB*100X hyperactive transposase to transduce human cord blood CD34^+^ cells with DsRed and a hybrid IHK–β-globin transgene. IHK transduced cells were successfully differentiated into multiple lineages which all showed transgene integration. The mature erythroid cells had an increased β-globin to γ-globin ratio from 0.66±0.08 to 1.05±0.12 (*p = *0.05), indicating expression of β-globin from the integrated *SB* transgene. IHK–β-globin mRNA was found in non-erythroid cell types, similar to native β-globin mRNA that was also expressed at low levels. Additional studies in the hematopoietic K562 cell line confirmed the ability of cHS4 insulator elements to protect DsRed and IHK–β-globin transgenes from silencing in long-term culture studies. Insulated transgenes had statistically significant improvement in the maintenance of long term expression, while preserving transgene regulation. These results support the use of *Sleeping Beauty* vectors in carrying an insulated IHK–β-globin transgene for gene therapy of sickle cell disease.

## Introduction

Hematopoietic stem cells (HSCs) are an attractive target for genetic modification to treat diseases such as sickle cell anemia, β-thalassemia, and severe combined immunodeficiency (SCID), among others [1], [2]. The ability of HSCs to be harvested with relative ease, withstand *ex vivo* manipulation and their long established clinical use in transplants allows for numerous potential gene therapy strategies. This is supported by a recent report of successful treatment of a β-thalassemia patient using a recombinant hemoglobin-expressing lentivirus to transduce autologous CD34^+^ cells [3]. However, as in other viral vector clinical trials, significant and potentially problematic clonal expansion was observed.

HSCs give rise to a diverse number of cell types and maintain their ability to self-renew [4], [5]. The current methods for gene transfer in clinical trials rely principally on the use of modified retroviruses such as Moloney murine leukemia virus (MMLV) and lentivirus, which have been known to insert into actively transcribed genes and potentially promote oncogenesis [6]–[8]. The development of leukemia in 4 out of 20 patients enrolled in a trial of MLV-based therapy for X-SCID continues to be a cautionary example to the field of gene therapy [9], [10].

Non-viral vectors offer a more easily implemented and potentially safer method to genetically modify cells [11]–[13]. Non-viral vectors, however, are challenged by relatively low gene transfer efficiency and the difficulty of maintaining long-term stable expression [13]–[15]. Transposons can be utilized to provide an integration mechanism for stable copy number and long-term expression important for non-viral gene therapies [16]–[19]. The *Sleeping Beauty* (*SB*) transposon system has become increasingly useful in fields ranging from cancer biology and stem cell research to gene therapy over the last decade since its resurrection from the salmonid genome [17], [20], [21]. The recent development of the *SB*100X transposase and its utility in modifying primary CD34^+^ cells are evidence of the system's continued improvements and potential clinical significance [22], [23].

In this study, we used the *SB* transposon to develop an optimal expression system for erythroid-specific gene expression in specific lineages [24]. Typical viral vectors drive erythroid-specific β-globin expression using 2.7 kilobases (kb) from the β-globin locus control region and promoter [25]. This large size makes the approach impractical for the *SB* system, which loses efficiency in a linear fashion from 2-kb cargos up to a practical limit of 6–10 kb [16], [22]. The 1-kb erythroid promoter IHK can provide high-level expression of β-globin in hematopoietic cells [26], [27]. The IHK promoter is composed of the *ALAS2* intron 8 strong erythroid enhancer, the HS40 core element upstream from the ζ-globin gene, and the *Ankryin-1* promoter [27].

Even with improved integration efficiency and transgene design, the *SB* transposon system is still subject to epigenetic changes in the host genome such as DNA methylation, and histone modification [28]–[30]. In a recent report, the heterologous chicken HS4 β-globin insulator elements (cHS4) flanking a fluorescent transgene were employed in *SB* vectors and provided protection against progressive silencing of transposon integration sites [21]. This chicken HS4 insulator element was able to block enhancer activity, eliminating or diminishing the influence of enhancer elements on genes in close proximity [31], [32]. In addition, it has been shown to protect transgenes from silencing by epigenetic modifications such as CpG methylation when the transgene is flanked on both sides by cHS4 insulator elements [33], [34]. Furthermore, this enhancer blocking activity of the insulator may mitigate the potential transactivation of neighboring host genes by *SB*-mediated transgene insertion, which has been observed in a few insertion loci in previous studies [28], [35].

In this study, the IHK-β-globin gene was used in combination with the *SB*100X transposase system to provide erythroid-specific expression of β-globin when integrated into primary CD34^+^ cells, and differentiated into multiple lineages. In addition, our results confirmed previous reports that the incorporation of insulator elements into the *SB* construct(s) protects transgenes against long-term inactivation, possibly from epigenetic changes, which reduce expression of integrated transgenes over time [21], [35]. Together, the results support the potential use of a *SB* IHK–β-globin expression system for the gene therapy of sickle cell disease in human clinical trials.

## Results

### *SB*100X transduces CD34^+^ cells in *cis* or *trans*

**Figure 1:**
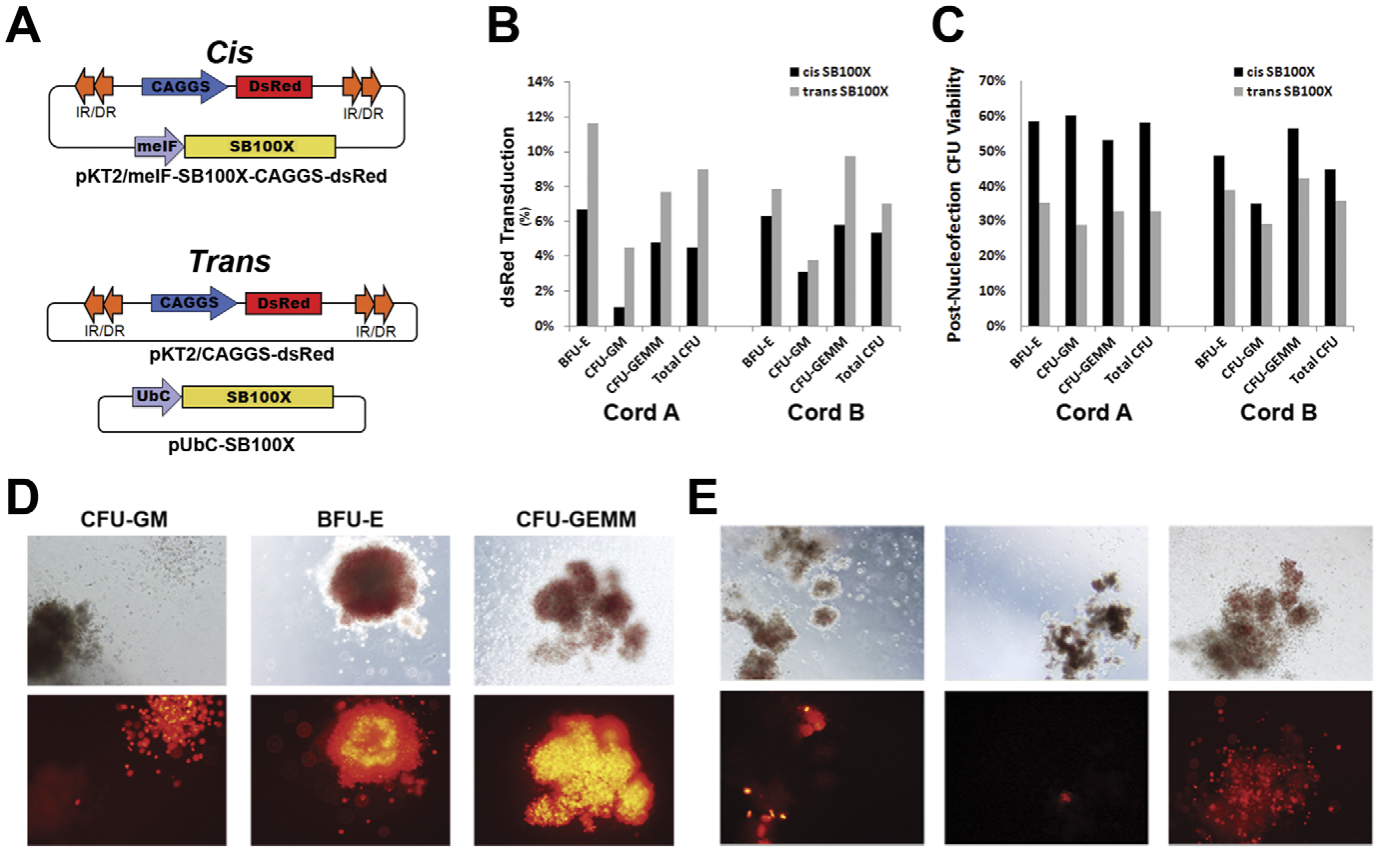
*SB*100X Transduction of CD34^+^ cells in *cis* and *trans* configurations. (**A**) Schematics of *cis* and *trans Sleeping Beauty* plasmid vectors (not to scale). (**B**) Percentage of DsRed^+^ CFUs in *cis* or *trans*. (**C**) Colony Forming Units (CFU) derived from CD34^+^ cells nucleofected with 10 µg of *cis* pKT2-meIF-*SB*100X-CAGGS-DsRed or 10 µg of *trans* pKT2/CAGGS-DsRed and 5 µg of UbC-*SB*100X using split cell samples. Totals from 4 replicates per vector per cord were normalized to non-nucleofected CFU output to give CFU viability. (**D**) Colonies were scored by standard light microscopy (150× magnification) for CFU type (top row) and epifluorescence microscopy for DsRed expression (bottom row). DsRed^+^ cells were found among CFU-GM (left panels), BFU-E (center panels), and CFU-GEMM (right panels). *Cis* constructs shown. (**E**) DsRed^+^ colonies were not homogeneous in expression patterns suggesting position effect variegation and epigenetic changes after transgene integration.

To test the utility of a *cis SB*100X construct, 5×10^5^ CD34^+^ cells from freshly isolated cord blood were mock-nucleofected with no DNA, nucleofected with either 10 µg of pKT2/CAGGS-DsRed, 10 µg of pKT2/CAGGS-DsRed plus 5 µg of UbC-*SB*100X, or 10 µg of pKT2/meIF-*SB*100X-CAGGS-DsRed and recovered for 2 hours (**Figure 1A**). Cells were evaluated in standard CFU assays. In the non-transposase pKT2/CAGGS-DsRed condition, we observed no DsRed positive colonies at 14–16 days and comparable viability to the *cis* plasmid post-nucleofection (data not shown). The ability of *SB*100X to transduce hematopoietic cells and the effect of nucleofected DNA on colony yields was assessed. The *cis SB*100X vector averaged 5% DsRed^+^ colonies compared with 8% for the *trans SB*100X vector (**Figure 1B**). Nucleofection resulted in DNA-induced toxicity with 51% viability with the *cis* vector and 34% in *trans* when normalized to mock-nucleofected controls (**Figure 1C**). DsRed^+^ cells were observed in BFU-E, CFU-GM, and CFU-GEMM cell types in both *trans* and *cis*, but not in the non-transposase controls (**Figure 1D**, *cis* shown; **Figures S1 and S2**). In addition, some single colonies displayed heterogeneous expression patterns (**Figure 1E**, *cis* shown; **Figure S3**), suggesting variable epigenetic changes.

### Nucleofected CD34^+^ cells differentiate *in vitro*

**Figure 2:**
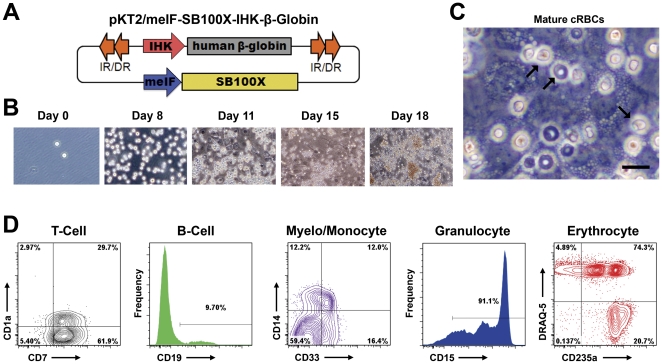
Nucleofected CD34^+^ cells retain their ability to differentiate *in vitro*. (**A**) Schematic of *cis* IHK–β-globin *SB* construct (not to scale). (**B**) Photomicrographs (100× total magnification) show the progression through 18 days of erythroid differentiation. (**C**) Erythroid differentiation can proceed through enucleation of mature cultured red blood cells (cRBCs); black arrows from left-to-right: nucleated erythroid, enucleated biconcave, and recently enucleated cup-shaped cells in culture at day 18 (400× total magnification; black scale bar is 10 µm in length). (**D**) Each differentiation trial was stained for respective markers of differentiation at 1∶100 dilutions in staining buffer. Gates were set using isotype controls or unstained controls as appropriate. A minimum of 5×10^3^ singlet events was collected for each sample. Granulocyte cultures were stained with CD15-PE-Cy7 (left panel); erythrocyte cultures with CD235a-FITC and DRAQ-5 at 1∶2000 (center left panel); B-cell cultures were stained for CD19-AlexaFluor700 (center panel); T-cell cultures with CD1a-FITC and CD7-AlexaFluor700 (center right panel); and myelocyte/monocyte cultures were stained with CD14-V450 and CD33-AlexaFluor700 (right panel).

To assess the erythroid specificity of the IHK promoter, we differentiated nucleofected CD34^+^ cells from cord blood into multiple lineages from both myeloid and lymphoid classes. CD34^+^ cells (7.5×10^5^ to 1×10^6^, >90% CD34^+^) obtained from three separate umbilical cords were nucleofected with 10 µg of pKT2/meIF-*SB*100X-IHK-β-Globin (**Figure 2A**). Cells were incubated for two hours in recovery media with cytokines and seeded into differentiation cultures for granulocytic, erythrocytic, B-cell, T-cell, and myelocyte/monocyte development. Differentiated cultures were assessed for granulocyte, erythrocyte, B-cell, T-cell, and myelocyte/monocyte development by identification of surface markers using flow cytometry (**Figure 2D**). Granulocytes showed robust differentiation with over 90% of CD15^+^ cells after ten days. In contrast, B-cell cultures demonstrated ∼10% CD19 expression at 3 weeks, consistent with previous reports on differentiation without specific enrichment [36]. T-cell cultures were associated with >90% CD7^+^ cells and 30% CD1a^+^/CD7^+^ cells at 4 weeks. Myelocyte/monocyte cultures showed just over 40% of cells expressing CD14, CD33, or both. Erythroid cultures demonstrated that >95% of cells expressed CD235a and ∼20% had enucleated by 18 days as determined by DRAQ5 staining. The erythroid cultures were followed from a low density seeding (day 0) through expansion and seeding onto a confluent MS-5 feeder layer (days 8 and 11, respectively) to maturation and hemoglobinization (days 15 and 18; **Figure 2B**). By day 18, nucleated erythroid cells, enucleated biconcave cells, and recently enucleated cup-shaped cells were apparent throughout the culture (black arrows left-to-right; **Figure 2C**).

### IHK-β-globin transgene mediates erythroid-specific hemoglobin expression

**Figure 3:**
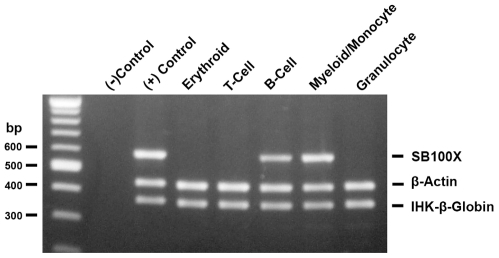
Transduced CD34^+^ cells retain IHK transgene after expansion and differentiation. Total genomic DNA of 60 ng was PCR-amplified to detect the presence of IHK–β-globin, *SB*100X, and β-actin control.

**Figure 4:**
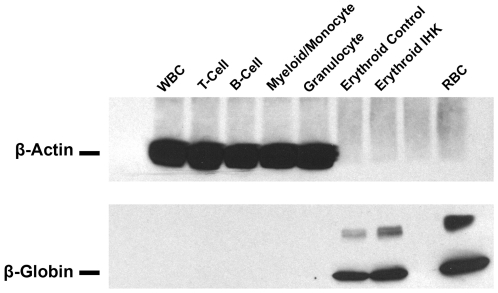
IHK-transduced CD34^+^ cells show β-globin expression only in erythroid cells. Thirty µg of total protein from IHK-transduced CD34^+^ cells differentiated into T-cells, B-cells, myeloid/monocytes, and granulocytes and 100 ng of total protein from non-transduced control or IHK-transduced CD34^+^ cells differentiated into erythrocytes were subjected to western blot using antibodies specific for β-globin and β-actin.

**Figure 5:**
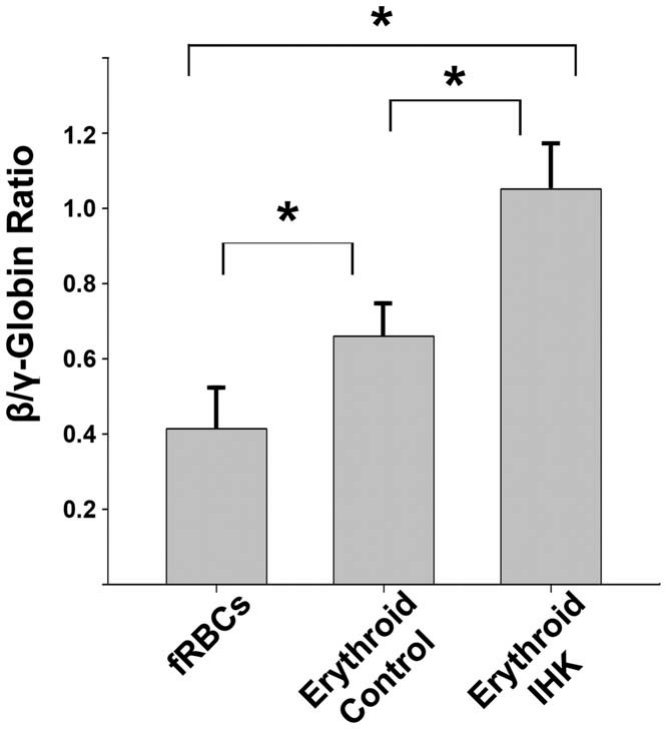
IHK-transduced CD34^+^ progenitors increase β-globin expression in erythroid progeny. Hypotonic lysates from fetal RBCs from the donor cords, erythrocytes from non-transduced CD34^+^ cells, and erythrocytes from IHK-transduced CD34^+^ cells were analyzed by RP-HPLC to determine relative quantities of individual globin chains. Data from three trials are presented as mean ± SEM, * indicates *p*<0.05, paired t-test.

To test IHK-β-globin transgene expression, purified genomic DNA, RNA, and protein was isolated from differentiated erythrocytes, granulocytes, myelocytes/monocytes, B-cells, and T-cells and examined for IHK–β-globin. The IHK–β-globin gene was detected in genomic DNA from all trials, while *SB*100X coding sequence was found in 10 of 15 trials using a highly sensitive PCR assay (**Figure 3**, trial 1). Optimization of multiplex genomic PCR demonstrated an ability to detect *SB*100X DNA down to 3 copies (**Figure S4**). In two of the three erythroid trials the *SB*100X-coding sequence was not detectable, likely attributable to a lower seeding density and a higher level of expansion compared to other lineages. Expression of β-globin in the differentiated populations was determined by western blotting with conditions optimized to detect as little as 3 ng of adult hemoglobin spiked into 50 µg of white blood cell lysate (**Figure S5**). Both IHK–β-globin transduced and control erythroid cultures showed robust β-globin expression, while expression was not detectable in the non-erythrocyte lineages (**Figure 4** **; Figures S6 and S7**). HPLC analysis of hemoglobin from control and IHK transduced-erythroid cells showed a significant increase (*p<*0.05) in the ratio of β-globin to γ-globin from 0.66±0.08 to 1.05±0.12 in the three IHK transduced erythroid cultures relative to the three control cultures (**Figure 5**).

### β-globin transcript is increased in non-erythroid progeny of IHK-transduced CD34^+^ cells while *SB*100X transcript is undetectable

**Figure 6:**
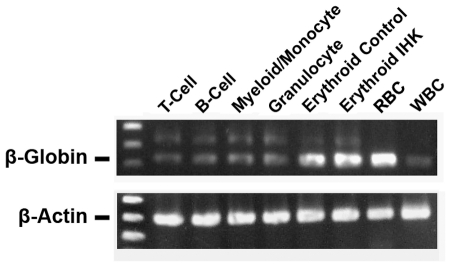
Increased β-globin transcript is detectable in non-erythroid progeny of IHK-transduced CD34^+^ cells. Twenty ng of total RNA was used for RT-PCR specific for spliced β-globin transcript and spliced β-actin. RNA from adult RBCs and erythrocyte-depleted adult WBCs was used as control.

RNA from differentiated cells derived from IHK-transduced CD34^+^ cells was subjected to RT-PCR using primers specific for β-actin and β-globin (**Figure 6** **; Figures S8 and S9**). All sets of samples showed similar levels of β-actin. Erythroid cells had increased β-globin transcript expression with the IHK-β-globin compared to control erythroid cells. Interestingly, the β-globin transcript was found in both the WBC control and at a higher level in the non-erythroid cells derived from IHK-transduced CD34^+^ cells. Because copies of *SB*100X-coding sequence were found to be carried in some transduced cells, RT-PCR for *SB*100X RNA was also performed which showed no detectable *SB*100X transcript (data not shown), thereby excluding the possibility of *SB* transposon mobility at this stage.

### Protection of transgene from progressive silencing by cHS4 insulator elements

**Figure 7:**
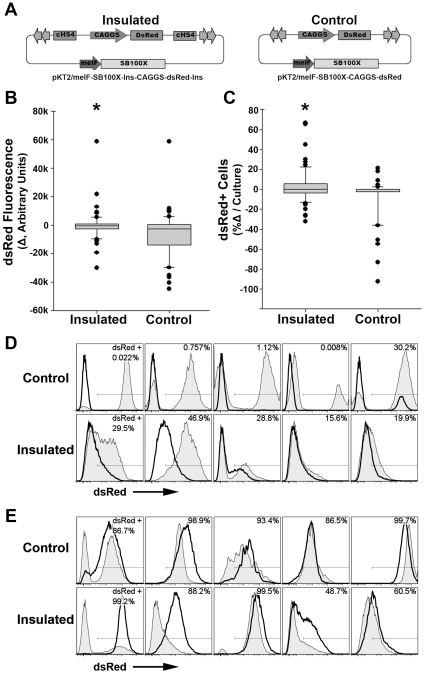
cHS4 insulator elements maintain long-term DsRed expression. (**A**) *SB* vectors insulated by cHS4 elements or non-insulated control *SB* vectors. CAGGS promoters with DsRed reporter transgenes and hyperactive *SB* transposase gene *SB*100X with meIF promoter are indicated. IR/DRs of *SB* are denoted by two short consecutive arrows. (**B**) Change of mean fluorescent intensity of DsRed between 2–4 weeks and 13–15 weeks after transfection displayed as a box plot showing 10^th^, 25^th^, 50^th^, 75^th^, and 90^th^ percentiles within each population with outliers shown individually. * indicates *p*<0.05, Mann-Whitney *U* test. (**C**) Change in the % DsRed^+^ cells in culture between 2–4 weeks and 13–15 weeks displayed as box plot. * indicates *p*<0.05, Mann-Whitney *U* test. (**D**) Examples of progressive silencing of DsRed demonstrated by profiles from the 5 clones with the greatest percent loss in DsRed expression for each vector. Shaded histogram, 2–4 weeks after transfection; solid histogram, 13–15 weeks after transfection. (**E**) Examples of positive shift of DsRed expression demonstrated by five clones with the greatest gains in DsRed expression for each vector term culture. Shaded histogram, 2–4 weeks after transfection; solid line, 13–15 weeks after transfection.

cHS4 insulator elements were introduced into the *SB* transposon vector system to test whether cHS4 insulator elements can provide protection against progressive silencing of the transgene [21], [28], [30]. The CAGGS-DsRed transgene cassette was cloned into a helper-independent *cis SB* vector containing the hyperactive *SB* transposase, *SB*100X, as pKT2/meIF-*SB*100X-Ins-CAGGS-DsRed-Ins (**Figure 7A**). In this construct, each boundary of the CAGGS-DsRed transgene cassette was flanked by a complete 1.2-kb cHS4 insulator element placed in parallel to limit potential homologous recombination between the two elements. K562 erythroid cells were transfected with pKT2/meIF-*SB*100X-Ins-CAGGS-DsRed-Ins or non-insulated control, pKT2/meIF-*SB*100X-CAGGS-DsRed. Two days after transfection, cells positive for DsRed were sorted into 96-well plates at a density less than 1 cell per well for both groups of transfected cells. Single-cell clones were isolated by either manual selection or FACSAria cell sorting. These clones were examined for the expression of DsRed on the 96-well plates, and DsRed-positive clones were further expanded to 24-well plates. Initially, 67 independent DsRed-positive clones from cells transfected with the insulated pKT2/meIF-*SB*100X-Ins-CAGGS-DsRed-Ins vector and 56 individual DsRed-positive clones from K562 cells transfected with non-insulated control pKT2/meIF-*SB*100X-CAGGS-DsRed vector were isolated by FACSAria or manual selection.

Between 2 and 4 weeks after transfection, each single-cell-derived clonal population was collected and analyzed for DsRed expression by flow cytometry. The expression of DsRed in clones was assessed once again at 13 to 15 weeks post-transfection, by using parameters normalized to the initial analysis at 2 to 4 weeks after transfection. Clones derived from cHS4-insulated *SB* constructs maintained the mean intensity of DsRed expression as well as the proportion of DsRed-positive cells at 13–15 weeks post-transfection when compared with the initial data obtained at 2–4 weeks. This was in contrast to clones from the non-insulated control *SB* vectors demonstrating long-term silencing as noted by significant decrease in the mean intensity of DsRed expression and percent DsRed^+^ cells (**Figures 7B and C**). While the majority of clones in both trials maintained relatively stable expression, comparison of clones showing the greatest losses and gains in percent DsRed^+^ cells for each vector demonstrated the potential for non-insulated transgenes to be highly down-regulated and insulated transgenes to gain expression over time. DsRed expression profiles of the 5 highest ranked clones for negative and positive shifts from each *SB* vector during long-term culture are compared (**Figures 7D and E**, respectively) while many clones showed little change between the time points.

**Figure 8:**
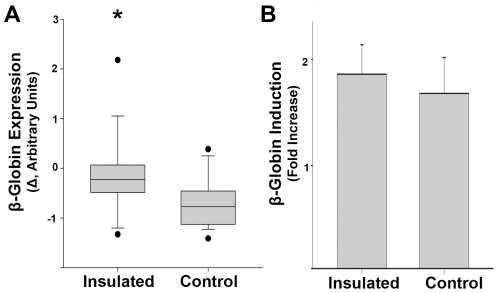
cHS4 insulator elements maintain long-term IHK–β-globin expression. (**A**) Change in β-globin expression from 4 to 14 weeks after transfection displayed as a box plot showing 10^th^, 25^th^, 50^th^, 75^th^, and 90^th^ percentiles within each population with outliers for the insulated pKT2/meIF-*SB*100X-IIβgI (n = 18) and non-insulated pKT2/meIF-*SB*100X-Iβg (n = 20) clones. * indicates *p*<0.001, paired t-test. (**B**) Fold increase in β-globin expression in response to hemin induction for insulated (n = 17) and non-insulated (n = 23) clones. Data shown as mean ± SEM, difference between groups was not significant, *p = *0.62.

To test the effect of the insulators on long-term β-globin transgene expression, we transfected K562 cells with the cHS4-insulated β-globin-expressing vector pKT2/meIF-*SB*100X-IIβgI and the non-insulated control pKT2/meIF-*SB*100X-Iβg. Two days after transfection, single cells were sorted manually and cultured in 96-well plates for two weeks, transferred to 24-well plates for 2-weeks and analyzed for genomic IHK by PCR. For IHK^+^ clones, hemin and non-hemin treated samples were collected at 4 and 14 weeks. Quantification of β-globin protein expression in hemin-induced samples at both time points showed a significant (*p*<0.001) loss of expression in the non-insulated vectors relative to the insulated vectors (**Figure 8A**). The change represents a mean loss of 13.1±81.6% of β-globin expression in the insulated group and of 66.1±50.3% of β-globin expression in the non-insulated group. In summary, the results were similar to those generated with the insulated and non-insulated *SB* vectors for DsRed. To investigate the effect of the insulators on IHK–β-globin regulation we tested the response of clones in each group to induction with hemin, which stimulates hemoglobin production and upregulation of erythroid-associated genes [37]. Induced clones from the insulated and non-insulated groups showed no significant differences (1.88±1.29 fold and 1.77±1.75 fold increases, respectively) in β-globin expression upon hemin induction (**Figure 8B**).

## Discussion

In this study we have shown that primary human CD34^+^ cells can be transduced with the *SB* construct, pKT2/meIF-*SB*100X-IHK-β-globin, can express hemoglobin in mature erythroid progeny, while maintaining tight, erythroid-specific expression. Previous studies have shown that IHK is potentially a more active promoter than the β-globin LCR, albeit in a viral vector. The small size of IHK relative to β-globin LCR/promoter-based vectors makes it well suited for the *SB* system as a mechanism to drive hemoglobin expression [22], [27]. It is important to reduce the overall size of the *SB* transgene cargo to promote efficient transposition and to lower the number of insertion sites necessary to provide therapeutic expression [22]. An interesting feature of IHK is its use of erythroid enhancer and promoter elements, which are not directly involved in normal β-globin production. While the IHK acts as a strong promoter in erythroid cells, we have previously shown that the native introns and untranslated regions of the β-globin gene are crucial for high-level expression of protein [26].

Analysis of the transgene content and expression of pKT2/meIF-*SB*100X-IHK-β-globin transduced cells showed that each of the nucleofected trials retained the IHK transgene in each lineage after 18 to 28 days of differentiation. In this study, as previously shown, we did find a low-level of transcription in non-erythroid cells, which was translationally suppressed and failed to produce detectable β-globin [26]. In that the inserted transposon still contains IR/DRs necessary for transposition, the potential remobilization of the transposon is a significant concern. We found that *SB* transposase-coding DNA can be detected at low levels within a transduced population but does not express detectable mRNA as determined by RT-PCR, consistent with a recent study of *SB* transduced cells [38].

In order to achieve maximum efficiency of transposon insertion into the host genome, we employed an enhanced transposase, *SB*100X, which was shown to be ∼100 times as active as the original version of the *Sleeping Beauty* transposase [39]. Stable gene transfer efficiencies of almost 50% were achieved with *SB*100X in the human cells differentiated from transfected hematopoietic CD34^+^ cells in remarkable contrast to stable gene transfer efficiencies of the original *SB* transposase (0.2 to 1%) or of second-generation hyperactive *SB*11 [16]. These previous results support the potential use of *SB*100X as a gene transfer agent for human CD34^+^ hematopoietic cells, and further development of the *SB* system in general.

We have also shown that cHS4 insulator elements can mitigate the tendency for *SB*-inserted transgenes to be inactivated over the long-term [30]. Long-term stable expression of a therapeutic transgene is critical in the successful gene delivery for genetic diseases such as β-thalassemia or sickle cell anemia. There are numerous reports of transgene silencing associated with delivery by either retroviral vectors or non-viral *SB* vectors [30], [40]. Most of this long-term inactivation of transgenes can be attributed to epigenetic changes such as DNA methylation or chromatin modifications in the inserted transgenes [29], [30], [41]. The chicken HS4 insulator element employed here has previously been shown to block enhancer activity, eliminating or diminishing the influence of enhancer elements on genes in close proximity whether the enhancer is included within the transgene or placed close to the insertion site in the host genome [31], [32]. In addition, the cHS4 insulator is known to protect transgene from silencing by epigenetic modification such as CpG methylation when the transgene is flanked by cHS4 insulator elements [33], [34].

In this study, cHS4 insulator elements successfully in protected both CAGGS-DsRed and IHK–β-globin transgenes from progressive silencing in the context of *SB* transposon system. Importantly, we showed that a cHS4-flanked transgene prevented sharp inactivation of the transgene and in some cases increased transgene expression over the ∼14-week culture period. The results are consistent with a previous report in which the insulators prevented long term silencing of a yellow fluorescent protein transgene integrated into host genome by *SB* [21]. This is especially important in potential gene therapies where increasing vector copy numbers to compensate for inactivation increases the risk of harmful mutagenesis [14]. Insulators also play a role in preventing local changes in gene expression due to insertion sites. The appropriate regulation of an insulated IHK–β-globin transgene is also important for potential therapies and in this study we found no significant differences in transgene expression upon differentiation between K562 cells transduced with insulated or non-insulated vectors. Insulators combined with the erythroid-specific IHK promoter could potentially confer long-term therapeutic levels of hemoglobin for the treatment of sickle cell disease, β-thalassemia, and other hemoglobinopathies utilizing CD34^+^ cells.

This combination of a highly efficient non-viral vector, a cargo protected from epigenetic changes, and a tightly regulated transgene is potentially of great utility in a clinical setting. Further *in vitro* and *in vivo* studies are needed to evaluate the pKT2/meIF-*SB*100X-IIβgI and variant vectors in primary hematopoietic cells over long term experiments and the impact of such vectors on disease models. Expanding on the results of this study will lead to the development of a vector that will be suitable for clinical trials.

## Methods

### *Sleeping Beauty* transposon constructs

The *cis* pKT2-meIF-*SB*100X and *trans* pKT2-RV *SB* transposon vectors were produced using standard molecular cloning techniques (**Figure 1**). The hyperactive *SB*100X transposase gene was driven by the constitutive murine eukaryotic initiation factor 4A1 promoter (Invitrogen, Carlsbad, CA) [22]. In order to protect the transgenes from long-term silencing, we constructed *SB* transposon vectors containing two insulator elements flanking multiple restriction endonuclease recognition sites derived from pIRES2-EGFP (Invitrogen) [31], [42]. Chicken HS4 insulator elements (1.2 kb) were transferred to the BamHI and SacI sites of pIRES2-EGFP from the plasmid DNA construct, pJC13-1 (kindly provided by Dr. Gary Felsenfeld, NIDDK, NIH), by digestion of pJC13-1 with XbaI or SacI, respectively [31]. Ends of the released insulator elements and digested pIRES2-EGFP vectors were modified to be compatible with the ligation. After successful transfer of two cHS4 insulator elements into pIRES2-EGFP, a DNA fragment encompassing two cHS4 insulator elements and a partial multicloning site (MCS) between the insulators was cloned into pKT2-meIF-*SB*100X or pKT2-RV *SB* vectors by modification of DNA ends with Klenow, T4 DNA polymerase and Antarctic Phosphatase (New England Biolabs, Ipswitch, MA). This resulted in the insulated *SB* vectors, pKT2/meIF-*SB*100X-Ins-MCS-Ins or pKT2/RV-Ins-MCS-Ins. The two insulators encompassing the partial multicloning site were inserted in the same, parallel direction to minimize loss of transgenes by recombination. As a final step, the 3.2 kb IHK-β-globin transgene fragment (**Figure S10**) was derived from pT2/IHK-β-globin//eIF-*SB*10 by digestion with PstI, and introduced into the partial MCS of pKT2/meIF-*SB*100X-Ins-MCS-Ins and pKT2/RV-Ins-MCS-Ins. These constructs are hereafter referred to as pKT2/meIF-*SB*100X-IIβgI and pKT2/RV-IIβgI, respectively. Control *SB*-IHK-β-globin constructs with no insulator elements were made by cloning the 3.2-kb IHK-β-globin transgene fragment into pKT2-meIF-*SB*100X and pKT2-RV vectors by EcoRV digestion and blunt-end ligations (pKT2/meIF-*SB*100X-Iβg and pKT2/RV-Iβg, respectively).

*SB* vectors of cHS4-insulated CAGGS-DsRed fluorescent transgene cassette were constructed with pKT2/meIF-*SB*100X-Ins-MCS-Ins and pKT2/RV-Ins-MCS-Ins in a similar manner. CAGGS-DsRed transgene was derived from digestion of pCAGGS-DsRed with SpeI and HindIII, where DsRed transgene was obtained with PCR from pDsRed-Express (Clontech, Mountain View, CA) and cloned into pCAGGS vector [43]. The CAGGS-DsRed transgene fragment was then blunted by Klenow (New England Biolabs) and cloned into SalI recognition site of pKT2/meIF-*SB*100X-Ins-MCS-Ins and pKT2/RV-Ins-MCS-Ins, making pKT2/meIF-*SB*100X-Ins-CAGGS-DsRed-Ins and pKT2/RV-Ins-CAGGS-DsRed-Ins, respectively. Non-insulated control vectors, pKT2/meIF-*SB*100X-CAGGS-DsRed and pKT2/RV-CAGGS-DsRed, were generated by directly cloning SpeI-HindIII CAGGS-DsRed transgene into original pKT2-meIF-*SB*100X and pKT2-RV, via blunt-end ligation.

### K562 cell culture, transfection, and isolation of single-cell clones

K562 (ATCC catalog number CCL-243) cells were maintained in RPMI 1640 (Invitrogen) supplemented with 10% FBS (Omega Scientific, Tarzana, CA). Eighteen hours prior to transfection, 2×10^6^ cells were freshly plated in 100-mm Petri dishes in RPMI 1640 medium without antibiotics. Four µg of plasmid DNA (pKT2/meIF-*SB*100X-Ins-CAGGS-DsRed-Ins or non-insulated control vectors, pKT2/meIF-*SB*100X-CAGGS-DsRed) was transfected using Lipofectamine 2000 (Invitrogen) according to the manufacturer's instructions. Cells were passaged to fresh medium after 18 hours and DsRed^+^ single-cell clones were sorted into 96-well plates via FACSAria (BD Biosciences, San Jose, CA) or manual isolation under epifluorescence microscopy. Single-cell clones were then transferred to 24-well plates and maintained for long-term (≥12 weeks) studies.

K562 cells were also transfected with pKT2/meIF-*SB*100X-IIβgI and pKT2/meIF-*SB*100X-Iβg as above and PCR screening of clones for the IHK sequence and confirmation of β-globin expression at 4 weeks after transfection was used to select clones for long-term culture. β-globin expression was induced with 20 mM hemin added to the culture media for 72 hours prior to collection for western blot analysis.

### Western blot analysis

For analysis of K562 derived clones, western blot analysis was conducted as previously described [26]. Nitrocellulose blots were stained simultaneously with mouse antibodies against human β-globin (sc-21757; Santa Cruz Biotechnology, Santa Cruz, CA) and β-actin (AC-15; Sigma-Aldrich, St. Louis, MO) at 1∶1000 dilutions. Blots were visualized using HRP-conjugated goat anti-mouse secondary antibody (32430; Thermo Scientific, Rockford, IL), Super Signal Dura substrate (34076, Thermo Scientific), and X-ray film (BioMax; Eastman Kodak, Rochester, NY). For analysis of CD34^+^ derived cells, western blot analyses were conducted as described previously [26], [44]. Pre-cast SDS-PAGE gels (Mini-PROTEAN TGX 4–20%; Bio-Rad, Hercules, CA) were loaded with differentiated cell lysates, resolved at 100 volts, and cut into upper and lower sections with the aid of a pre-stained ladder at 25–30 kDa (GE Healthcare, Little Chalfont, United Kingdom). The upper portion was transferred and analyzed for β-actin as previously described [26]. The lower portion was transferred and analyzed for β-globin using a modified technique as reported [44]. The modified transfer for β-globin was performed for 20 min at 30 volts and the membranes were fixed in 1× PBS with 0.4% paraformaldehyde for 30 min. Normal adult human white blood cell lysate and red blood cell lysate were used as controls.

### Gel densitometry

Western blots of IHK–β-globin-transduced K562 clones were analyzed using established guidelines [45]. Briefly, multiple exposure lengths of each blot were scanned using an MP160 document scanner at highest resolution (Canon, Tokyo, Japan). Images were quantified with FIJI v1.45 software (fiji.sc) using the gel analysis tool set to integrate OD without calibration or background correction. β-globin to β-actin ratios for all exposures from each trial were calculated and the median of all measurements was used as a measure of β-globin expression levels.

### Nucleofection of CD34^+^ cells

Cord blood mononuclear cells (MNCs) were separated using Ficoll-Paque PLUS density medium (GE Healthcare). The mononuclear fraction was subjected to ACK buffer (150 mM NH_4_Cl, 10 mM KHPO_4_ and 0.1 mM EDTA, pH 7.4) lysis to remove any residual red blood cells. Cells were washed three times using 1× PBS containing 0.5% BSA and 2 mM EDTA (all Invitrogen); and CD34^+^ cells were separated using Miltenyi MACS CD34 magnetic beads (Miltenyi Biotechnology, Bergisch Gladbach, Germany) according to the manufacturer's instructions. CD34^+^ purity was assessed using flow cytometry and was >90% in all cases (**Figure S11**). Cells were counted and resuspended at 0.75 to 1×10^6^ cells per 100 µl of CD34^+^ complete Nucleofection solution (Lonza, Basel, Switzerland). Cells were mixed with plasmid DNA and nucleofected using the Amaxa Nucleofector II with program U-008. Nucleofected cells were immediately removed into pre-warmed and equilibrated recovery medium consisting of X-VIVO 10 without phenol red or gentimycin (Lonza), 100 ng/ml Stem Cell Factor (SCF), 10 ng/ml IL-3, 10 ng/ml Flt-3L (all Invitrogen) and cultured in 6-well plates for 2 hours prior to differentiation.

### Differentiation of CD34^+^ cells

Erythroid differentiation was carried out on MS-5 stromal cells as previously described [46], [47]. Briefly, 2×10^4^ cells were plated in 2 mL serum-free expansion medium containing 100 ng/ml SCF, 5 ng/ml IL-3, 3 U/ml erythropoietin (EPO), and 1 mM hydrocortisone (Sigma-Aldrich) and cultured in 6-wells plates at 37°C and 5% CO_2_ for 4 days. The cells were then diluted into 6 ml of the same medium in a 25 cm^2^ flask and cultured for an additional 4 days. Expanded cells were washed with basal medium and resuspended in 25 ml of basal medium with 3 U/ml EPO and plated onto a newly confluent 75 cm^2^ flask of MS-5 feeder cells and cultured for 3 days. For final maturation, erythroid cells were co-cultured on feeder cells for 10 days using basal medium without cytokines with one medium change after 5 days at which time 2×10^6^ cells were harvested for nucleic acid purification. T-cell differentiation was carried on OP9 stromal cells as previously described [48]. Briefly, 5×10^4^ nucleofected cells were resuspended in αMEM (Invitrogen) with 20% FBS (Atlanta Biologicals, Lawrenceville, GA), 5 ng/ml IL-7, and 5 ng/ml Flt3L (both Peprotech, Rocky Hill, NJ) onto sub-confluent OP9-DLL1 cells. Fresh media with cytokines was replaced every 3 to 4 days. B-cell differentiation was carried out on MS-5 stromal cells as reported [36]. Briefly, 1.5×10^5^ nucleofected CD34^+^ cells were resuspended in 15 ml of αMEM with 10% FBS, 10 ng/ml SCF, and 10 ng/ml G-CSF and seeded onto a confluent MS-5 layer in a 75 cm^2^ flask. Ten milliliters of fresh medium was added after 7 days and cells were maintained with twice weekly half-media changes. Myeloid and granulocyte differentiations were performed on MS-5 stromal cells as described [23], [49]. Briefly, 10^5^ nucleofected CD34^+^ cells were seeded in 1 ml IMDM with 10% FBS, 2 mM L-alanyl-glutamine, 50 ng/ml SCF, and 50 ng/ml IL-3 in six-well plates. After 3 days in culture, cells were washed and seeded into 25 cm^2^ flasks in basal medium plus 50 ng/ml SCF, 50 ng/ml IL-3, and 10 ng/ml G-CSF. Cells were maintained at 2×10^5^ to 8×10^5^ cells per ml in complete medium with cytokines for 10 days until harvest.

### Colony forming assays

Colony forming assays were performed using MethoCult 4435 as recommended by the manufacturer (StemCell Technologies, Vancouver, Canada). Cells were seeded in quadruplicate and colonies were scored after 14–16 days using an Olympus IX70 epifluorescence light microscope (Olympus, Tokyo, Japan). Digital images were batch processed using ImageMagick 6.0 (imagemagick.org).

### Flow cytometry

Cell surface markers were characterized using fluorochrome-conjugated antibodies specific for human antigens: CD34-AlexaFluor647 (4H11; eBioscience, San Diego, CA), CD1a-FITC (HI149; BD Pharmingen), CD3-V450 (HIT3a; BD Horizon), CD7-AlexaFluor700 (M-T701; BD Pharmingen), CD14-V450 (MphiP9; BD Horizon), CD15-PE-Cy7 (HI98; BD Pharmingen), CD19-AlexaFluor700, CD33-AlexaFluor700, CD45-V500 (HI30; BD Horizon), CD45-PerCP-Cy5.5 (1D2; eBioscience), CD71-PE (OKT9; eBioscience), and CD235a-FITC (HIR2; eBioscience). Cells were labeled in staining buffer (1× Hanks Balanced Salt Solution without Ca^2+^ or Mg^2+^+2% FBS+1 mM EDTA) in antibody dilutions of 1∶100 at a concentration of 10^7^ cells per ml. Viability was assessed using 7-AAD (Southern Biotech, Birmingham, AL) or eFluor780 Fixable Viability Dye (eBioscience) and dead cells excluded from analysis. DNA content was assessed using DRAQ5 according to the manufacturer's instructions (eBioscience). Data acquisition was performed using a BD LSRII flow cytometer (BD Biosciences). Cell sorting was performed using a BD FACS Aria (BD Biosciences). Flow cytometric data was analyzed using FlowJo 7.6.4 software (Tree Star Inc).

### Sample collection

Cells were harvested and frozen in liquid nitrogen until processed. DNA, RNA, and protein were purified using the Qiagen AllPrep Mini kit (Qiagen, Valencia, CA). Briefly, progressive collection from sample lysate began with DNA collection using DNeasy columns, followed by RNA collection with RNeasy columns, and protein precipitation and washing using Buffer APP as recommended by the manufacturer. For HPLC analysis of hemoglobin, 1.5×10^7^ non-adherent erythroid cells were pelleted and lysed using 50 µl of HPLC grade water (Invitrogen); crude lysate was centrifuged at 18,000× *g*, and cleared lysate was frozen in liquid nitrogen and stored at −80°C prior to analysis.

### PCR analysis

*SB* and IHK DNA detection was done in a multiplex format using Qiagen HotStarTaq hot-start PCR system using primers IG2, BAG3, and SS9, for HS40/Ankyrin-1 junction in the IHK promoter, an Intron/Exon junction in β-Actin, and the *SB*100X coding sequence, respectively (**Table S1**). Sixty ng of genomic DNA was used from each test condition and the reaction was assembled according to the manufacturer's instructions and amplified using a two-phase cycle to promote amplification of low-levels of *SB*100X DNA (**Figure S4, Table S1**). RT-PCR for β-globin and β-actin was conducted as previously described [26]. Briefly, 20 ng of total RNA was amplified with primers specific for β-actin and β-globin using the Titan One Tube RT-PCR kit (Roche Applied Sciences, Indianapolis, IN) according to the manufacturer's instructions (Primers BAM1 and BGM1; **Table S1)**. RT-PCR for *SB*100X was performed using primer set SS9 which is specific for the *SB* coding sequence and 100 ng of total RNA.

### HPLC analysis

Globin chains were identified using a modification of the method described earlier [50]. An Agilent 1200 HPLC system (Santa Clara, CA) was used with a C4 column, a water/acetonitrile/trifluoroacetic acid gradient run at 0.7 ml/min at 23°C, and spectroscopic detection at 220 nm.

### Statistical analysis

Statistical significance of data was performed using SigmaStat 3.5 software (Systat Software, Chicago, IL).

## Supporting Information

Figure S1 **DsRed^−^ CFUs imaged with a bright field filter (top row), pan-fluorescence filter (middle row), and rhodamine fluorescence filter (bottom row).**
(TIF)

Figure S2 **Homogeneous DsRed^+^ CFUs imaged with a bright field filter (top row), pan-fluorescence filter (middle row), and rhodamine fluorescence filter (bottom row).**
(TIF)

Figure S3 **Heterogeneous DsRed^+^ CFUs imaged with a bright field filter (top row), pan-fluorescence filter (middle row), and rhodamine fluorescence filter (bottom row).**
(TIF)

Figure S4 **Sensitivity test of** ***SB*** **100X multiplex detection.** Sixty µg of normal human genomic DNA was spiked with 5000 copies of pKT2/IHK-β-globin plasmid and varying amounts of pUbC-SB100X plasmid and amplified as described in text.
(TIF)

Figure S5 **Western blot sensitivity test for β-globin.** Fifty µg of white blood cell lysate was spiked with varying amounts of adult hemoglobin from red blood cell hypotonic lysate, which was quantified directly using Drabkin's method at 540 nm. (**A**) Samples analyzed using modification of previously reported protocol [44]. (**B**) Samples analyzed using standard methodology [27].
(TIF)

Figure S6 **Western blot of IHK-β-globin trial 2.**
(TIF)

Figure S7 **Western blot of IHK-β-globin trial 3.**
(TIF)

Figure S8 **RT-PCR of IHK-β-globin trial 2.**
(TIF)

Figure S9 **RT-PCR of IHK-β-globin trial 3.**
(TIF)

Figure S10 **Schematic of the IHK–β-globin transposon components.**
(TIF)

Figure S11 **Assessment of CD34^+^ purity prior to nucleofection in IHK–β-globin trials.**
(TIF)

Table S1 **Primers and PCR cycles.**
(XLS)
