# Supplementary figures and images for: Erythroid-Specific Expression of β-globin from Sleeping Beauty-Transduced Human Hematopoietic Progenitor Cells
Source: PLoS One. 2011 Dec 28;6(12):e29110. doi: 10.1371/journal.pone.0029110 (PMC3247234; doi:10.1371/journal.pone.0029110)

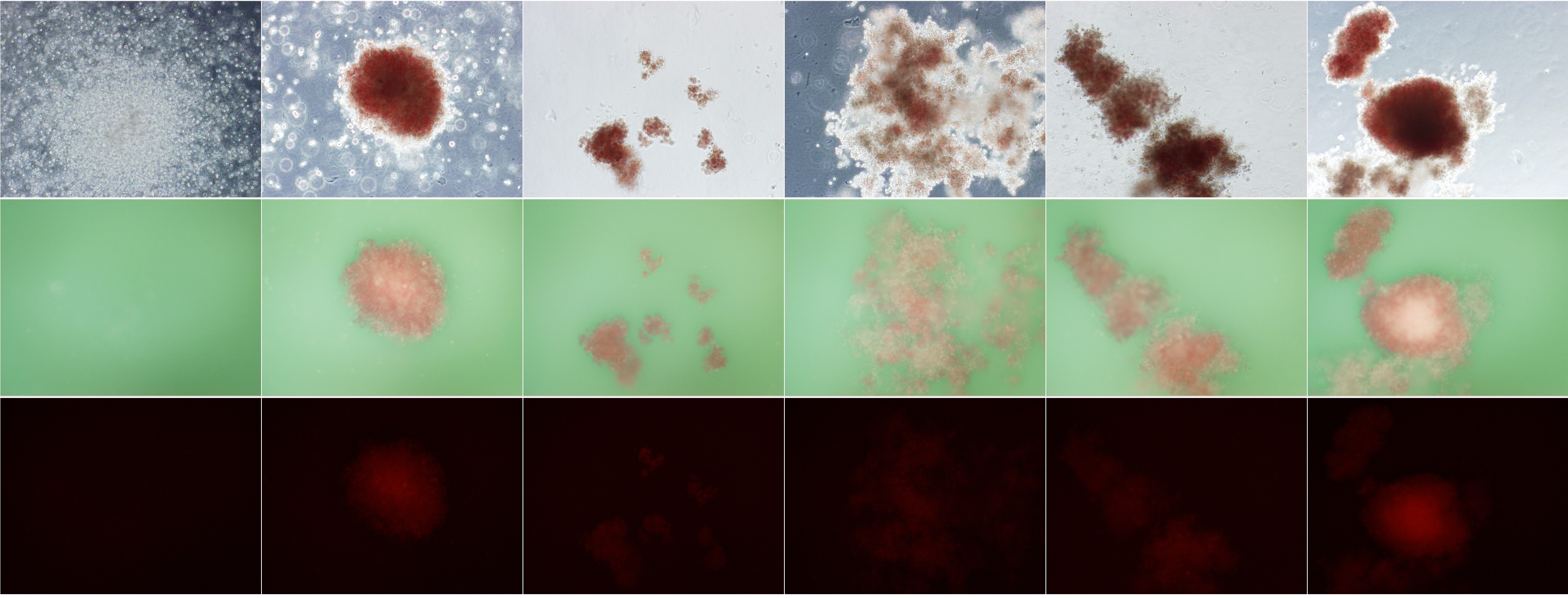

Supplement: Figure S1 — DsRed− CFUs imaged with a bright field filter (top row), pan-fluorescence filter (middle row), and rhodamine fluorescence filter (bottom row). (TIF) [file pone.0029110.s001.tif]

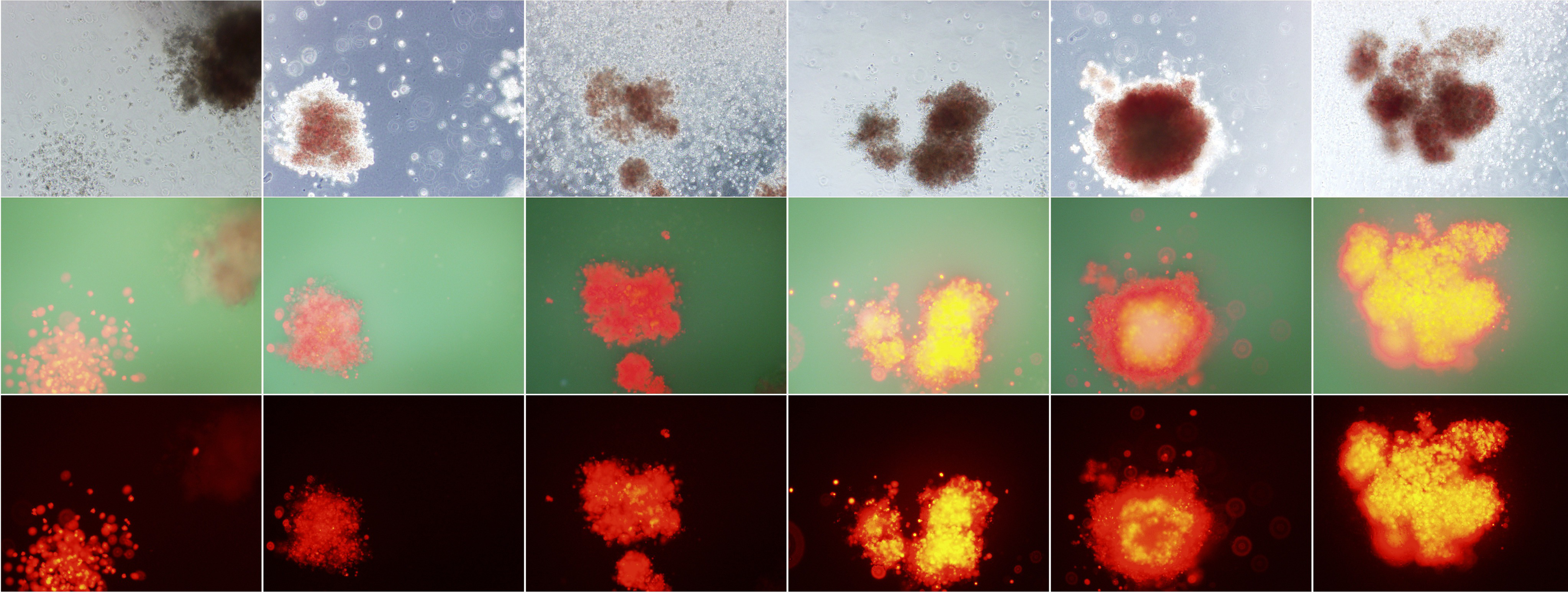

Supplement: Figure S2 — Homogeneous DsRed+ CFUs imaged with a bright field filter (top row), pan-fluorescence filter (middle row), and rhodamine fluorescence filter (bottom row). (TIF) [file pone.0029110.s002.tif]

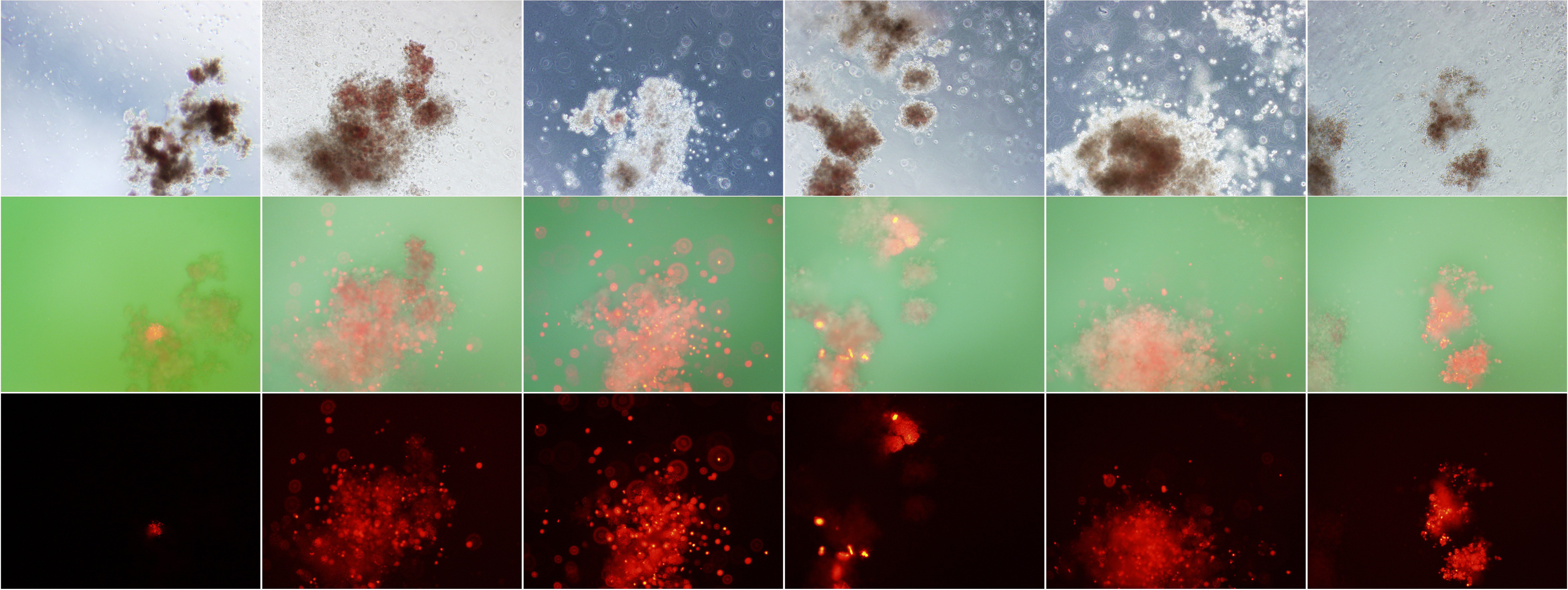

Supplement: Figure S3 — Heterogeneous DsRed+ CFUs imaged with a bright field filter (top row), pan-fluorescence filter (middle row), and rhodamine fluorescence filter (bottom row). (TIF) [file pone.0029110.s003.tif]

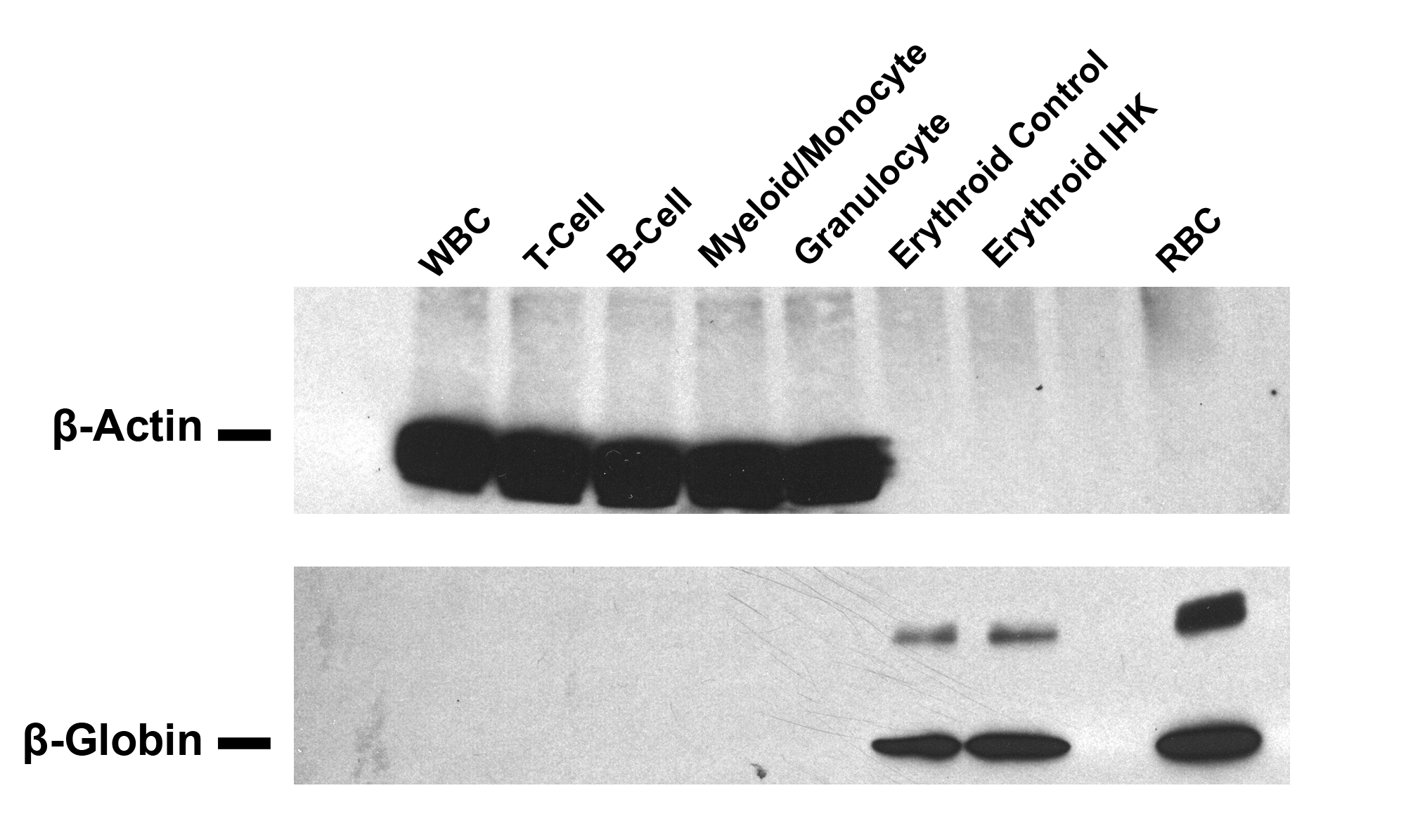

Supplement: Figure S6 — Western blot of IHK-β-globin trial 2. (TIF) [file pone.0029110.s006.tif]

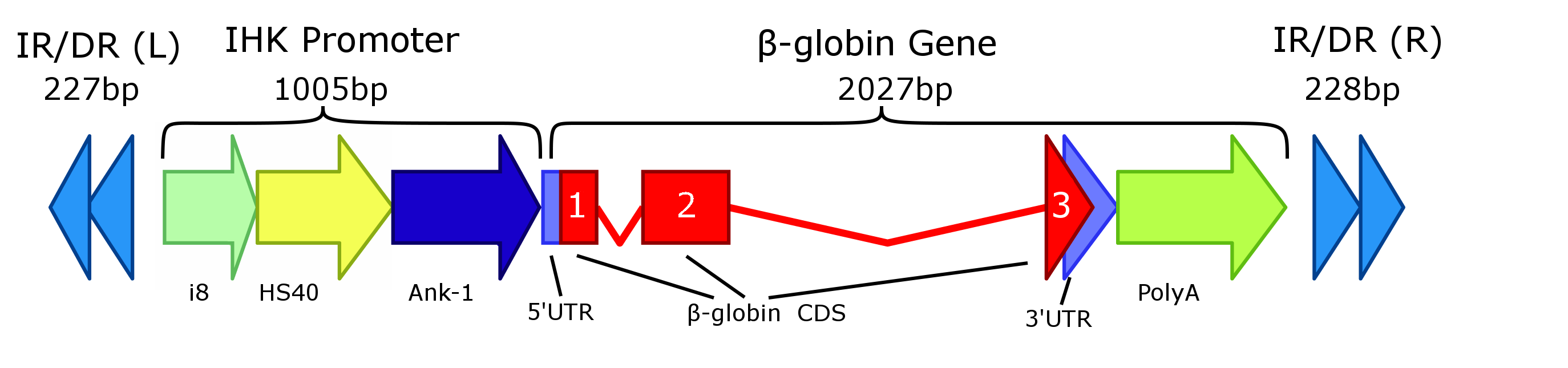

Supplement: Figure S10 — Schematic of the IHK–β-globin transposon components. (TIF) [file pone.0029110.s010.tif]

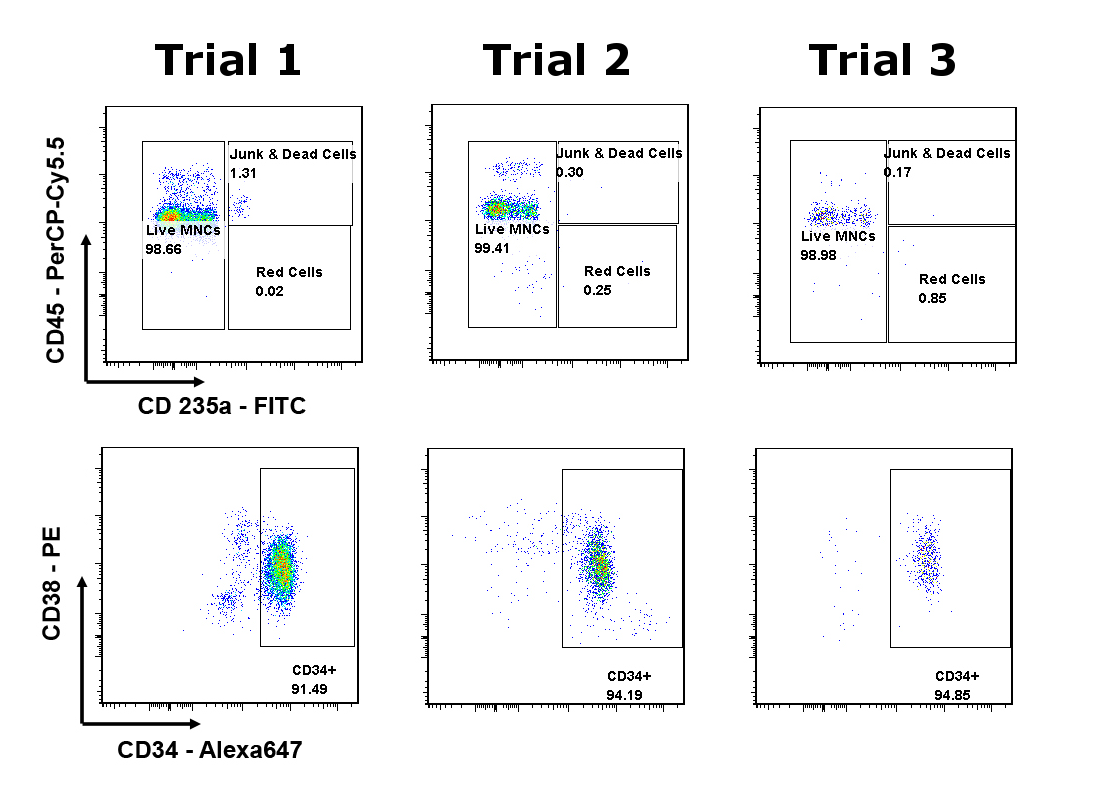

Supplement: Figure S11 — Assessment of CD34+ purity prior to nucleofection in IHK–β-globin trials. (TIF) [file pone.0029110.s011.tif]
